# Hæmorrhagic Pancreatitis

**Published:** 1906-05-19

**Authors:** 


### HÆMORRHAGIC PANCREATITIS.

A case of this rare condition is described by Drs. Wigg and Marten in the March issue of the "Australasian Medical Gazette." The patient was a man of 41 years, with a good record of health. He came under observation for symptoms suggesting the onset of influenza—namely, headache, pain in the loins and limbs, shivering, and loss of appetite. His temperature was 102° F. On the following day he was better, the fever having entirely disappeared. On the fifth day he returned to his business, but two days later became ill again, the symptoms being epigastric pain, with frequent vomiting of a green, slimy fluid. The temperature was normal, and the bowels had been slightly moved during the day. There was a slight degree of icterus, with tenderness over the epigastrium, but neither distension nor dullness about the belly. The urine contained a trace of bile, but no albumin. The case was at this stage regarded as one of gall-stones, and was treated with hot applications, calomel, and morphine. The next day brought an aggravation of the symptoms. Vomiting and hiccough were incessant, the jaundice more marked, and constipation complete. Things remained in this condition for two days longer, when a swelling was discovered below the right costal margin. It was about the size of a closed fist, dull on percussion, and very tender. Later in the day the swelling rapidly increased and extended downwards into the region of the right flank. It was decided that an operation was necessary. At this stage the patient was extremely ill: there was some general distension of the belly, this distension being most noticeable over the right hypochondriac region, where also there was great tenderness and an impairment of percussion resonance. The abdominal walls were rigid and moved but little on respiration.

When the belly was opened its cavity was found to contain a large quantity of brownish fluid. The gall-bladder appeared to be natural. The great omentum was much thickened, deeply injected, and curled up upon itself: it was, moreover, studded by small yellow spots of the size of miliary tubercles. The falciform ligament of the liver was covered with similar spots, and a few were visible upon the surfaces of the liver. A drainage-tube was inserted and the patient returned to bed. A small piece of the affected omentum which had been removed for examination was reported to be in a condition of fat necrosis due to an acute pancreatitis.

The patient continued to vomit incessantly, while the discharge of brownish fluid from the wound was profuse. The temperature was always low, and sometimes subnormal; the pulse was thready and rapid, and the respirations shallow. The patient died of exhaustion a week after the onset of symptoms. Throughout this period the vomiting and hiccough were constant and unrelieved by any medication except the liberal use of morphine. The discharge from the wound changed its character, and became offensive; its smell was like that of a partially digested meal. This was due to the digestion of tissue in the neighbourhood of the pancreas, as was shown by post-mortem examination. The discharge from the wound caused great digestive destruction of the skin in the neighbourhood. Details of the post-mortem examination are not supplied.

In connection with the fat-necrosis, which is commonly associated with the hæmorrhagic and necrotic varieties of acute pancreatitis, Osler states that

Langerhans has produced fat-necrosis by the injection of pancreatic extract into the perinephric fatty tissues of dogs. Hildebrand and Dettmer have shown experimentally that the necrosis is due to certain constituents of the pancreatic juice, but not trypsin. This ferment is steapsin, and its presence has been demonstrated in certain instances of the disease by Flexner. This authority has also produced hæmorrhagic pancreatitis by the injection of artificial gastric juice into the pancreatic duct. Opie has also produced the condition by the injection of bile into the pancreatic duct of dogs, and has shown that the penetration of bile into the pancreas may be the cause of the disease in nature.

